# Insights into the Use of Point-of-Care Ultrasound for Diagnosing Obstructive Sleep Apnea

**DOI:** 10.3390/diagnostics13132262

**Published:** 2023-07-04

**Authors:** Alexandros Kalkanis, Dries Testelmans, Dimitrios Papadopoulos, Annelies Van den Driessche, Bertien Buyse

**Affiliations:** 1 Department of Respiratory Diseases, University Hospitals Leuven, KU Leuven, Campus Gasthuisberg, 3000 Leuven, Belgium; 2 Department of Endocrinology, General Hospital Rivierenland, Campus Bornem, 2880 Bornem, Belgium

**Keywords:** obstructive sleep apnea, ultrasound, screening

## Abstract

Obstructive sleep apnea (OSA) is a sleeping disorder caused by complete or partial disturbance of breathing during the night. Existing screening methods include questionnaire-based evaluations which are time-consuming, vary in specificity, and are not globally adopted. Point-of-care ultrasound (PoCUS), on the other hand, is a painless, inexpensive, portable, and useful tool that has already been introduced for the evaluation of upper airways by anesthetists. PoCUS could also serve as a potential screening tool for the diagnosis of OSA by measuring different airway parameters, including retropalatal pharynx transverse diameter, tongue base thickness, distance between lingual arteries, lateral parapharyngeal wall thickness, palatine tonsil volume, and some non-airway parameters like carotid intima–media thickness, mesenteric fat thickness, and diaphragm characteristics. This study reviewed previously reported studies to highlight the importance of PoCUS as a potential screening tool for OSA.

## 1. Introduction

Obstructive sleep apnea (OSA) is a common sleep-related breathing disease characterized by recurrent upper airway (UA) narrowing due to an imbalance between mechanical load and compensatory neuromuscular responses during sleep, resulting in sleep fragmentation and hypoxemia, as well as increased morbidity and mortality [1]. OSA is also a common risk factor for postoperative cardiopulmonary problems [2,3] with increased use of perioperative healthcare resources [4]. In OSA, the breathing disturbances are recurrent episodes of either complete blockage of UA (apnea) or substantially reduced UA diameter (hypopnea) while sleeping [5]. These recurrent events are associated with variable degrees of oxygen desaturation (SaO_2_) and hypoventilation, and such events are halted by brain stimulation to maximize the activity of UA dilator muscles to increase the UA diameter [6,7,8]. The severity of OSA is traditionally expressed using the apnea–hypopnea index (AHI). In mild OSA, the AHI is 5–15 per hour; in moderate OSA, it is between 15–30 per hour; and in severe OSA, the AHI is at least 30 or more per hour [9,10].

The underlying pathophysiology is complex, and it is the result of an interplay of four determinants: a narrowed/crowded collapsible upper airway, poor muscle responsiveness, high loop gain, and low arousal threshold. The impact of each determinant and the combination of determinants can be different and explain the existence of different phenotypes of OSA [11]. A structurally narrower and more collapsible UA is the most prominent feature of OSA severity, which can be characterized by an interaction between redundant soft tissue, decreased tone of the UA dilator muscles, and the bony anatomy of UA, which accounts for two-thirds of the heterogeneity in the AHI [12,13]. Not only is the UA narrower and more collapsible in OSA patients while awake, but it also collapses more easily during sleep as the activity of the UA dilator muscles decrease after sleep onset [14,15,16].

Detecting moderate-to-severe OSA is critical for preventing cardiovascular and cerebrovascular disorders, including potentially life-threatening cardiac problems during surgery. However, a large proportion of patients with OSA remain undiagnosed at the time of surgery [17]. Polysomnography (PSG) is the gold-standard laboratory technique to diagnose OSA, but it is costly and not commonly available [18,19]. Currently, screening for OSAS is being gradually implemented in the preoperative setting. The available screening techniques are mostly questionnaire-based, and although they are generally sensitive enough, these techniques are not specific [20,21] and do not have a high enough negative predicting value results [22,23]. Point-of-care ultrasound (PoCUS) has been used by anesthetists to efficiently evaluate UA [24]. This technique could also be useful for the diagnosis and screening of OSA together with other available methods because it is portable, painless, and has an increased specificity [24]. Specialized ultrasound tests have already been broadly and diversely utilized through PoCUS applications by anesthetists in the perioperative setting in order to guide patient management and improve clinical outcomes [25]. But only limited studies have evaluated the effectiveness of PoCUS as a screening tool for OSA.

This narrative review aimed to describe our current knowledge on the different applications of the PoCUS technique in the measurement of various anatomical parameters in OSA patients.

## 2. Materials and Methods

Studies were eligible for inclusion in the review if they evaluated patients with OSA compared to non-OSA controls for parameters measured with ultrasonography. OSA patients were required to have a positive sleep study (PSG or polygraphy), while non-OSA controls were required to have a normal one. We also retained studies without a control group if ultrasound parameters were compared between different categories of OSA severity. While we focused on the imaging of the UA, we also included studies that evaluated non-airway parameters as a screening tool for OSA. However, we excluded echocardiography and liver ultrasound measurements because of their low specificity for the diagnosis of OSA. There were no exclusion criteria based on the type and brand of the ultrasound devices or the ultrasonographic modes and probes used in the studies. Any measurement obtained by ultrasonography, such as dimensions, moving distance, or signal intensity, either in an awake or a sleep state was eligible as an outcome. Only published observational and randomized controlled studies in the English language were included. Studies were grouped according to the outcomes measured (UA versus non-UA anatomical parameters).

To find relevant studies, the “Google Scholar” and “PubMed” online databases were searched in December 2022. The search strategy included keywords related to OSA and ultrasonography: (“sleep apnea*” OR “sleep-disordered breathing” OR “sleep-related breathing” OR OSA) AND (ultrasound* OR ultrasonography* OR sonography*). The titles and abstracts of the retrieved records were then screened to determine eligibility for inclusion. The full texts of eligible studies were obtained, and the following data were extracted: participants’ characteristics, ultrasound parameters measured, patients’ state of consciousness during measurement, and results comparing OSA to controls or comparing between different OSA severity categories. No synthesis was undertaken because of the limited available evidence and the heterogeneity in the ultrasonographic measurements. Instead, the data are presented narratively and in a tabular format. Images were obtained using a portable Philips Lumify ultrasound in a healthy volunteer to provide visualization of the most commonly used techniques for the scanning of UA anatomical parameters.

## 3. Results

The data extracted from the included studies were classified according to the outcomes into UA and non-airway parameters and were subsequently tabulated (Table 1). A more thorough narrative explanation, along with some technical standards and images, are presented in this section.

### 3.1. Ultrasound of the Upper Airway

As mentioned before, OSA is characterized by recurrent periods of upper airway occlusion during sleep. Some OSA patients can present with clinically obvious skeletal anatomic variations such as micrognathia or retrognathia. Additional clinical physical evaluation of the upper airway region is focused on the tongue’s size and its hiding of the soft palate and tonsil size.

When examining the structures of the upper airway, its anatomical variations and possible predisposition for OSA [26] have been an early target for radiographic studies. There are several significant limitations to conventional cephalometrics with standardized lateral radiograph of the head and neck. Moreover, a meta-analysis of this method showed a limited value and only one cephalometric variable, mandibular body length, has a clinically meaningful correlation with the severity of sleep apnea [27]. This knowledge has recently been revised in the latest systematic review and meta-analysis on anatomical determinants as mandibular length is not considered an important factor for OSA; the primary anatomical determinants of upper airway mechanical stability are hyoid position, tongue size, pharyngeal length, and obesity measures [28]. Three-dimensional magnetic resonance imaging (MRI) has also been suggested for the identification of craniofacial risk factors for obstructive sleep apnea [29], but it brings the question of cost-effectiveness. Even though ultrasound of the upper airway has already been broadly used in airway management, it is only recently that Osman et al. presented anatomical points of sonographic interest for sleep apnea patients [30].

#### 3.1.1. Tongue Parameters (Figure 1 and Figure 2)

The tongue is one of the most common anatomical locations for an obstruction of the upper airways, blocking them through its increased volume and/or a fallback position [31]. One of the most frequently used methods in the practice of sleep medicine as a first step to clinically identify patients at a high risk for sleep apnea is the Friedman tongue position (FTP) [32], relative to the tonsils/pillars, uvula, soft palate, and hard palate. Non-echographic imaging studies investigating tongue anatomy in OSA patients have mainly focused on tongue volume, fat distribution, and muscle activity [33]. The high cost and the radiation of computed tomography (CT) and magnetic resonance imaging (MRI) discourage the scaling of the use of these imaging techniques in sleep apnea patients.

**Figure 1:**
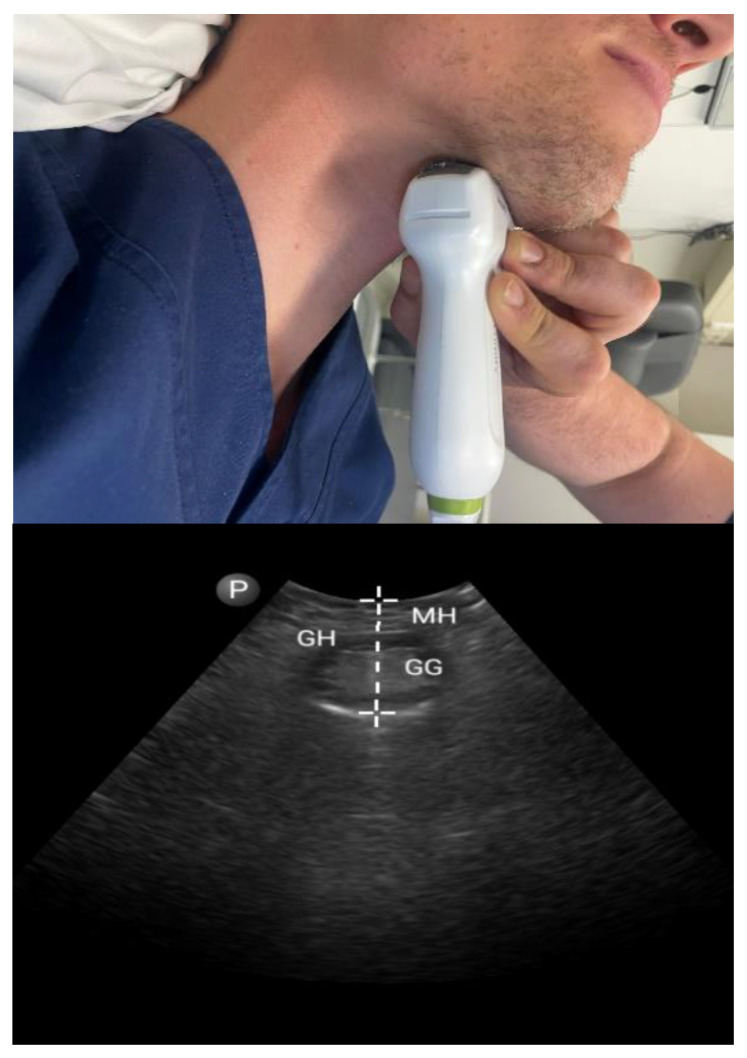
Submental approach in coronal view: coronal mid-tongue base thickness. Mylohyoid muscle (MH), geniohyoid muscle (GM), and genioglossus muscle (GG).

**Figure 2:**
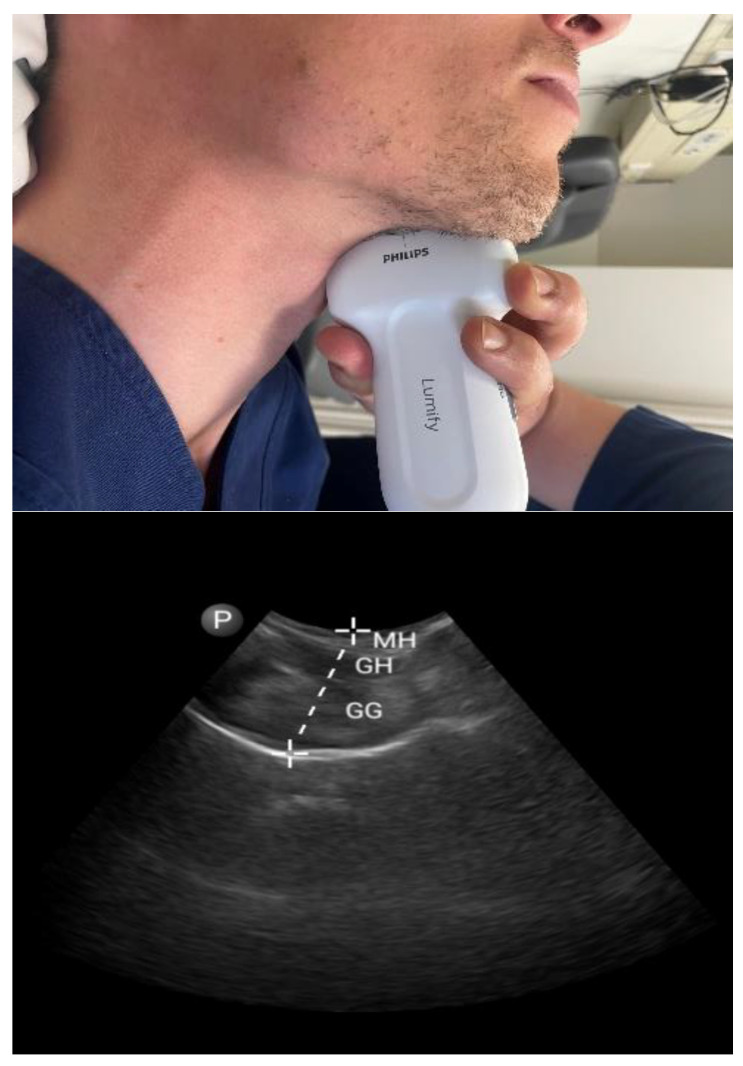
Submental approach in sagittal view: sagittal mid-tongue base thickness. Mylohyoid muscle (MH), geniohyoid muscle (GM), and genioglossus muscle (GG).

Yu et al. used ultrasonography to quantify tongue fat in OSA patients [34]. Their study was carried out with a basic hypothesis that a higher amount of tongue fat would result in a higher echo intensity and, consequently, OSA patients would exhibit a higher echo intensity of ultrasonics. The results indicated that OSA patients tended to have a higher tongue fat percentage depicted in ultrasound, which was also supported by MRI analysis. The amount of tongue fat in OSA cases was higher in both fat and lean patients in comparison to the controls, suggesting that tongue fat is a potential predictor of OSA [34].

Besides this qualitative evaluation of the tongue, researchers have studied several other markers and dimensions in this anatomical region. Total sagittal thickness (TSag) (submental skin to tongue dorsum), tongue muscle sagittal thickness (TMSag), submental fat sagittal thickness (SFSag), and submental fat transverse thickness (SFTrans) parameters did not show significant differences during awake and drug-induced sleep states [35]. On the contrary, when comparing US measurements at rest and during Müller’s maneuver (MM), significantly higher diameters and tongue volumes were detected in patients with OSA, both at rest and during MM, compared to the control group [36]. Chen et al., in an earlier study, performed a US quantitative assessment of the retroglossal airway in OSA patients and concluded that the thickness of the tongue base during MM is a potential indicator for OSA severity [37]. 

Another anatomical marker that is often studied along with the dimensions of the muscle itself is the distance between the two lingual arteries (DLA). Lahav et al. used an ultrasonic technique to explore the mucosa, connective tissues, muscles, and blood vessels of 41 male participants [38]. The analysis suggested that a distance of more than 30 mm between the lingual arteries increases the chances of OSA. This study also observed a correlation between BMI and OSA; however, the correlation was not as strong as that of the lingual distance of the vessels. In the aforementioned study by Abuan et al. [35], DLA elongated significantly during drug-induced sleep and had a positive correlation with AHI and BMI. 

More recently, Hussein et al. conducted a study on 90 participants, including both obese and non-obese patients. Parameters like DLA, retro lingual diameter (RLD), and tongue base thickness (TBT) were measured [39]. The results indicated that DLA and TBT were significantly higher and RLD was significantly lower in OSA patients than in the control. In addition, the increase in DLA and TBT was directly related to the severity of OSA, suggesting that both parameters could be useful for diagnosing the intensity of the disease. Lun et al. also studied ultrasonic anatomical characteristics of the oropharynx and looked for potential markers of OSAS. Among several measurements, lingual height, the anatomical distance between the mylohyoid muscle and the palate, was a significant factor for determining the severity of OSAHS patients independent of age, sex, and BMI [40].

By exploiting ultrasound’s capability for dynamic measurements of muscle contraction, Kwan et al. found good consistency and agreement between ultrasound and MRI in measuring posterior tongue displacement in healthy patients and patients with obstructive sleep apnea [41]. Manlises et al. evaluated both tongue dimensions and movements. Their results indicated that tongue movements were severely restricted in moderate-to-severe OSA cases, but a bidirectional motion could still be observed in mild OSA cases. Moreover, significantly higher tongue values were detected during MM and OSA patients had a higher mid-sagittal tongue area compared to the controls [42]. 

Arens et al. investigated the use of ultrasound shear-wave elastography (US-SWE) as a quantitative tool to measure tongue muscle activation during selective hypoglossal stimulation therapy (sHNS) and found significant differences during contraction only on the side of stimulation [43]. Another group used a novel ultrasonographic marker, the mean hyoid bone excursion (HBE), to not only visualize the response to treatment but also to assist the programming of hypoglossal nerve stimulation [44]. Recently Curado et al. used ultrasound to determine whether changes in tongue morphology under selective hypoglossal stimulation in OSA were associated with alterations in airway patency during sleep when specific portions of the hypoglossal nerve were stimulated. The authors demonstrated that HNS-induced responses in tongue morphology during wakefulness showed specific traits in ultrasound and correlated with airway patency responses during sleep [45].

This ability to quantify the morphologic changes of the tongue, together with movement in three dimensions, gives sHNS a more targeted character. Objective measurements of tongue muscle movement and activation undoubtedly present some difficulties due to the lack of a well-established technique, but these non-invasive methods provide new possibilities to distinguish and characterize responders from non-responders in hypoglossal nerve stimulation therapy as a sort of pre-treatment screening. Moreover, simultaneous ultrasonographic evaluation of changes in tongue morphology under selective hypoglossal nerve therapy can act as guidance for the optimal positioning and adjustment of the electrodes. 

#### 3.1.2. Tonsils

Enlarged tonsils can result, through airway obstruction, in difficulties in breathing, thereby frequently leading to snoring and sleep breathing disorders. Enlarged tonsils caused by tonsillitis are especially common in children who suffer from sleep apnea and are one of the indications for tonsillectomy [46]. Less frequently, enlarged tonsils can also cause sleep apnea or snoring in adults as well [47,48].

In the past, but also in more recent studies, different scales and anatomical criteria, like Brodsky’s grading scale [49,50], Archimedes’ principle [51], and the Ellipsoid formula [52], have been applied to estimate the size of the tonsils, especially in children who suffer from snoring and apneas. However, novel and emerging techniques based on ultrasonics are considered the most accurate and efficient [53]. Ultrasonics can be used in both children and adults for measuring tonsils. For instance, Mengi et al. measured the size of tonsils in 50 children and 35 adults before and after tonsillectomy [54]. In both children and adults, the ultrasonics method had a moderate correlation with Friedman grading, which is a standard clinical assessment of tonsil size. In a recent systematic review of PoCUS for screening OSA in the pediatric population, it was concluded that tonsil volume, calculated using ultrasound, correlated with surgical specimens, but not with OSA [55].

#### 3.1.3. Pharyngeal Parameters (Figure 3)

It has been proposed that a predominant anatomical component driving airway narrowing in apneic people is the thickness of the lateral parapharyngeal muscle walls, as shown on MRI [56]. Liu et al. used a systematic protocol for performing sonographic measurements targeting lateral pharyngeal wall thickness (LPWT) [57]. Their method showed a reproducibility of 90%, and LPWT correlated fairly to moderately (r = 0.37) with OSA severity even after correction for demographic variables. However, the authors reported that sonographic methods could overestimate LPW size, in comparison to magnetic resonance imaging (MRI)-based techniques.

**Figure 3:**
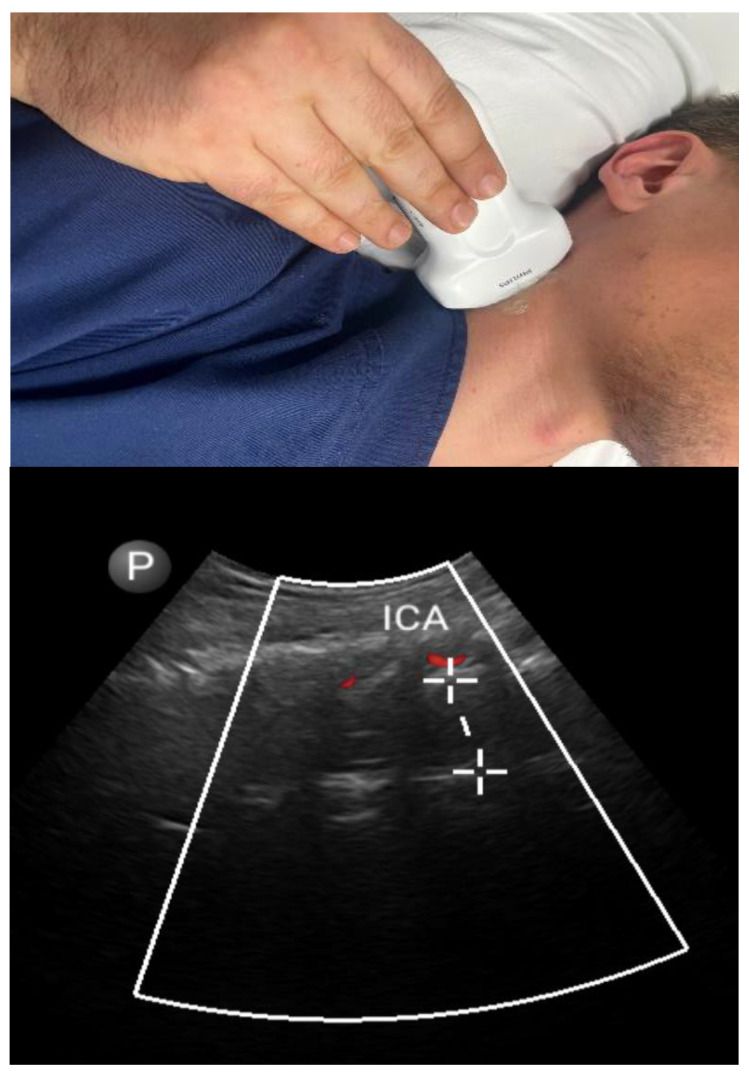
Lateral approach: lateral parapharyngeal wall thickness. Internal carotid artery (ICA).

In a more recent study, Molnár et al. used a combination of US LPWT values and anthropometric parameters to achieve a 93% effectiveness in OSA prognostication in one hundred patients [58]. In children, Yuen et al. compared the efficiency of ultrasonic techniques with MRI in measuring LPW size [59]. Their study consecutively recruited 34 children of ages 6 to 11. The results indicated a correlation of 0.72 between the measurements taken via ultrasonic and MRI-based techniques, indicating a strong agreement between the two methods in the oblique plane.

Hussein et al. showed a statistically significant increase in the lateral parapharyngeal wall thickness (LPWT) in OSA patients compared to controls [60]. Similarly, a statistically higher distance between lingual arteries (DLA) was also observed in OSA patients. In addition, OSA patients showed a significant decrease in retropalatal pharynx transverse diameter (RPD). The value of this latter ultrasonographic marker was shown in an older study: the diameters of the retro-glossal (RG) and retro-palatal (RP) regions were measured via submental US upon expiration during tidal breathing, forced inspiration, and Müller’s maneuver (MM) in one hundred and five consecutive referrals for suspected OSA [61]. Compared to non-OSA and mild-to-moderate OSA patients, those with severe OSA had a narrower RP diameter in all three maneuvers. 

### 3.2. Non-Airway Parameters

Along with airway parameters, some non-airway parameters, including the thickness of carotid intimal media and mesenteric fat, the diameter of the brachial artery (BA), flow dynamics of peripheral blood, ocular blood flow, and diaphragm, have also been studied for their association with OSA through ultrasound. 

#### 3.2.1. Carotid Intima–Media Thickness

Numerous studies have demonstrated the association of OSA with an increased risk of cardiovascular diseases. An increase in carotid intima–media thickness is an independent marker of atherosclerosis. Apaydin et al. used B-mode ultrasonography and found that increased carotid intima–media thickness was correlated with OSA [16]. In another study comprising 156 OSA patients, Ciccone et al. investigated the correlation between OSA duration and severity with carotid intima–media thickness through ultrasound. In patients with severe and long-lasting OSA, the value of carotid intima–media thickness was greater compared to controls [62]. In an ultrasonographic study performed by Altin et al., it was observed that the value of carotid intima–media thickness was significantly higher in severe OSA patients compared to moderate OSA patients [63]. Wattanakit et al. reported a significant connection between increased carotid intima–media thickness measured using ultrasound and OSA severity; but multivariate adjustments for demographic and metabolic characteristics reduced the correlation [64]. These studies supported the association between OSA and carotid intima–media thickness and suggest the value of an ultrasound screening test as a diagnostic marker for OSA. 

Chami et al. measured the diameter of the brachial artery and peripheral blood flow dynamics using flow-mediated dilation [65]. The severity of OSA was found to be positively correlated with increased brachial diameter. However, no positive correlation was observed between OSA and flow-mediated dilation. 

#### 3.2.2. Adipose Tissue

Previous research has revealed that higher visceral fat deposition is crucial for determining the risk of OSA [66,67], and the quantity of visceral fat is evaluated using cross-sectional imaging, such as MRI and CT. A significant association (r = 0.8) was observed between mesenteric fat thickness (MFT) evaluated by ultrasonography and total visceral adiposity measured by MRI in a pilot investigation of 37 Chinese patients [68]. According to a more recent publication by Liu et al., a positive correlation was found between MFT and OSA after adjusting for factors like age, sex, BMI, neck circumference, and preperitoneal and subcutaneous fat thickness [69]. Interestingly, they found that MFT, rather than the apnea–hypopnea index, was the major independent determinant of metabolic syndrome in individuals with suspected OSA, particularly in non-obese patients. This study showed that ultrasound is a useful tool for measuring mesenteric fat thickness, which means that it could also serve as a more specific testing method for the connection between OSA and metabolic disorders. 

Çetin et al. revealed, through ultrasonic analysis, that abdominal fat index (AFI) correlates with AHI and both increase with increasing severity of OSA [70]. Therefore, both indices can be combined in OSA predictions. Their study included 104 individuals (73 males and 31 females) aged 23 to 73. AFI was calculated as the ratio (Pmax/Smin) between maximum preperitoneal fat thickness (Pmax) and minimum subcutaneous fat thickness (Smin). Previously, Tokunaga et al. also calculated this ratio in OSA patients using MRI [71]. However, measuring the ratio using ultrasonics was considered by the authors as more accurate and easier to implement. 

Recently, Molnar et al. studied the predictive role of ultrasonographic-measured subcutaneous adipose tissue in the pathogenesis of OSA with the use of artificial intelligence; the presence of upper airway obstruction in drug-induced sleep endoscopy was compared to ultrasound measurements of subcutaneous adipose tissues (SAT) in the regions of the neck, chest, and abdomen. Among these measurements, the thickness of the chest SAT was of the same importance as BMI in the prognosis of OSA, and the thickness of abdominal SAT measured 2 cm above the umbilicus was shown to be the most helpful prognostic parameter. It should be noted that while submental adipose tissue was anatomically vital in upper airway obstruction, the analysis showed a limited role in the prediction of OSA [72]. This agrees with the results of a previous study, which reported that subcutaneous fat in the neck region and parapharyngeal fat in the airway vicinity were not correlated with the presence of OSA [73]. 

#### 3.2.3. Diaphragmatic Parameters

Pazarlı et al. measured diaphragm thickness in OSA patients [74]. Thickness was measured as the distance between the peritoneum and the pleura. This study included 108 individuals (67 males and 41 females). The results indicated that diaphragm thickness was significantly higher in OSA patients in comparison to controls during both inhalation and exhalation. Moreover, diaphragm thickness also correlated (r = 0.4) with the severity of the disease. Recently, Molnár et al. developed an algorithm based on diaphragm parameters [75]. The authors also studied movement and further developed a statistical tool to predict OSA. Their study included 100 patients, with 64 confirmed cases of OSA. The authors observed that OSA patients had significantly different thicknesses of the right and left hemidiaphragm compared to controls. Moreover, the changes in diaphragm thicknesses were positively correlated with AHI. Further, based on diaphragm thickness, age, sex, and some other demographic variables, the authors developed a logistic regression algorithm to predict OSA with sensitivity and specificity values of 91% and 81%, respectively. 

**Table 1:** Summary of important studies that reported the use of point-of-care ultrasound in obstructive sleep apnea.

Ref	Individuals	State of Consciousness	Ultrasound Parameters	OSA Outcomes	Main Findings
Airway Parameters					
[34]	83 adults	Awake	Tongue echo intensity	AHI	Tongue echo intensity was significantly associated with higher AHI (adjusted rho = 0.27).
[35]	26 adults	Awake and drug-induced sleep	Tongue parameters	AHI	During drug-induced sleep, the tongue muscles become thinner and the space between the two lingual arteries is significantly widened. The latter had a significant positive correlation with AHI (r = 0.51).
[36]	100 adults	Awake	Tongue parameters	OSA (AHI ≥ 5/h)	In the prognosis of OSA, a US sensitivity of 94% and a specificity of 91% were detected.
[37]	40 adults	Awake	Tongue base thickness	OSA (AHI ≥ 5/h)	Tongue base thickness during Müller’s maneuver was an independent predictor of OSA (OR = 2.11, 95% CI: 1.15, 3.87).
[38]	41 male adults	Awake	Tongue parameters	Moderate-to-severe OSA (AHI > 15/h)	Distance between lingual arteries >30 mm had a sensitivity of 80% and a specificity of 67% for diagnosing moderate-to-severe OSA.
[39]	90 adults	Awake	Tongue parameters	AHI	DLA and TBT were positively correlated with AHI.
[40]	171 adults	Awake	Tongue parameters	OSA severity (mild, moderate, and severe)	Lingual height was an independent predictor of OSA severity (OR = 1.14, 95% CI: 1.04, 1.24).
[41]	42 adults	Awake	Tongue movement	-	Agreement between MRI and ultrasound of posterior tongue displacement during inspiration.
[42]	56 adults	Awake	Tongue parameters and tongue movement	OSA (AHI ≥ 5/h)	OSA patients had a larger midsagittal tongue area and restricted movement of the tongue muscles.
[43]	18 adults	Awake	Ultrasound shear-wave elastography	-	Median shear-wave velocity increased during selective hypoglossal stimulation therapy.
[44]	17 adults	Awake	Tongue movement (hyoid bone excursion)	Response after HNS (reduction in AHI > 50% and AHI < 20/h)	HBE > 0.85 cm had a sensitivity of 83.3% and a specificity of 80.0% in predicting response after HNS.
[45]	12 adults	Awake	Tongue parameters and tongue movement	Change in airflow during sleep	Tongue protrusion with preservation of tongue shape predicted increases in patency during selective hypoglossal stimulation therapy.
[54]	50 children and 35 adults	Awake	Tonsil’s size	-	A high correlation was observed between ultrasound measurements and Friedman’s parameters.
[57]	76 adults	Awake	LPW thickness	AHI	LPWT correlated fairly to moderately with OSA severity (r = 0.37) but could lead to overestimation.
[58]	100 adults	Awake	LPW thickness	OSA	A combination of US measurements of LPW and anthropometric parameters had a sensitivity of 93% and a specificity of 94% for the detection of OSA.
[59]	34 children	Awake	LPW thickness	-	Ultrasound-based estimations were similar to MRI.
[60]	43 adults	Awake	LPW thickness and other upper airway parameters	OSA (AHI ≥ 5/h)	OSA patients had increased LPWT (sensitivity of 100%, specificity of 60%) and DLA (sensitivity of 90.9%, specificity of 60%), with a decreased RPD (sensitivity of 54.5%, specificity of 100%).
[61]	105 adults	Awake	Upper airway parameters	Severe OSA (AHI ≥ 30/h)	Change in retropalatal diameter during Müller’s maneuver and neck circumference had a sensitivity of 100% and a specificity of 65% for predicting severe OSA.
Non-airway parameters					
[16]	87 adults	Awake	Carotid arteries	OSA (AHI ≥ 5/h)	Significant association between OSA and carotid intima–media thickness.
[62]	156 adults	Awake	Carotid arteries	AHI and years from symptom onset	Association between OSA severity (r = 0.51) and duration (r = 0.34) and carotid intima–media thickness.
[63]	30 adults	Awake	Carotid arteries	OSA (AHI ≥ 5/h)	Association between OSA severity and carotid intima–media thickness.
[64]	985 adults	Awake	Carotid arteries	RDI	Weak correlation between respiratory disturbance index and carotid intima–media thickness.
[65]	682 adults	Awake	Branchial artery	AHI and % TST with spO_2_ < 90%	Baseline brachial artery diameter was significantly associated with both the apnea–hypopnea index and the hypoxemia index.
[69]	149 adults	Awake	Adipose tissue	AHI	Positive correlation of AHI with mesenteric (r = 0.43) and preperitoneal (r = 0.3) fat thickness.
[70]	104 adults	Awake	Adipose tissue	AHI	Statistically significant correlation between AFI and AHI (r = 0.23).
[72]	100 adults	Awake	Subcutaneous adipose tissue	OSA (AHI ≥ 5/h)	Anthropometric data, blood test parameters, and US SAT measures had a sensitivity of 100% and a specificity of 91.7% for predicting OSA.
[74]	108 adults	Awake	Diaphragm	AHI	Diaphragm thickness was higher in OSA patients and positively correlated with disease severity (r = 0.41 for end-expiratory, r = 0.45 for end-inspiratory).
[75]	100 adults	Awake	Diaphragm	OSA (AHI ≥ 5/h)	A combination of diaphragmatic dimensions, diaphragm dilation, age, sex, and BMI predicted the presence of OSA with 91% sensitivity and 81% specificity.

OSA, obstructive sleep apnea; AHI, apnea–hypopnea index; US, ultrasound; OR, odds ratio; CI, confidence interval; DLA, distance between lingual arteries; TBT, tongue base thickness; MRI, magnetic resonance imaging; HNS, hypoglossal nerve stimulation; HBE, hyoid bone excursion; LPW, lateral pharyngeal wall; RPD, retropalatal pharynx transverse diameter; RDI, respiratory disturbance index; TST, total sleep time; AFI, abdominal fat index; SAT, subcutaneous adipose tissue; BMI, body mass index.

## 4. Discussion

OSA is a sleep disorder that affects individuals of all ages and genders. Accurate and timely diagnosis are important aspects as OSA is associated with several other serious diseases such as cardiorespiratory disorders [76].

The first solid indication for a possible need to screen the population for OSA came through the results of the Wisconsin Sleep Cohort Study (WSCS), a National Institutes of Health (NIH)-funded epidemiologic research project conducted by the University of Wisconsin’s Specialized Center of Research in Cardiopulmonary Disorders of Sleep [77].

This study monitored 1522 randomly selected individuals for two decades to establish the total societal burden of sleep-disordered breathing. Mild OSA was reported in 17% of adults, and 6% of adults had moderate-to-severe OSA. 

A recent publication of the US Preventive Services Task Force (USPSTF) recommendation statement on screening for obstructive sleep apnea has been a systematic effort to address the high public health risk of untreated patients and sets a steppingstone for future research [78]. A combination of screening questionnaires, such as the Epworth Sleepiness Scale, the STOP questionnaire (snoring, tiredness, observed apnea, and high blood pressure), the STOP-Bang questionnaire (STOP questionnaire plus body mass index, age, neck circumference, and gender), the Berlin Questionnaire, and clinical factors, such as body mass index, neck circumference, and blood pressure, were proposed as a set of tools to be used by primary care providers. The purpose is to try to identify asymptomatic individuals or individuals with underrecognized symptoms. Further attention is currently being paid in research to specific groups of individuals who are more directly vulnerable to undiagnosed sleep apnea and who would benefit from a more thorough evaluation and a targeted intervention.

Professional drivers are such a population who show a higher prevalence of OSA compared to the general population, given that drivers with OSA show an increased risk for car accidents [79]. A retrospective review of all commercial driver medical examinations reported an overall positive OSA screening yield of 20.1% [80].

Patients on a surgical list are another target population. As previously mentioned in this manuscript, a careful screening during preoperative assessment along with possible initiation of appropriate treatment could assist in avoiding respiratory and cardiovascular complications and improve clinical outcomes [81].

Apart from the use of specialized ultrasound tests by anesthetists in these preoperative settings, PoCUS has already been broadly and diversely utilized in a wide range of specialties for the evaluation of different systems and pathologies [82].

Several studies have reported the use of ultrasound to measure the association of OSA with different airway and non-airway parameters. However, limited scientific work has been conducted so far to evaluate the specificity and effectiveness of PoCUS as OSA screening in a larger population. The systematic review and meta-analysis by Singh et al. in 2018 evaluated the utility of surface ultrasound measurements and identified several airway and non-airway parameters with moderate to good correlation with OSA diagnosis in the general population [83]. In the pediatric population, Burns et al. recently conducted a systematic review of PoCUS for screening of OSA. They also came to a positive conclusion regarding the correlation of ultrasonographic markers with other well-established reference measurements [55]. However, as mentioned by the authors, most of the results were based only on a small subset of studies and the increased heterogeneity decreased the generalizability and application of some of the PoCUS methods in screening settings. 

Moreover, we must be aware that ultrasound examination has not yet been evaluated for the measurement of other determinants of the pathophysiology of sleep apnea, such as loop gain and arousal threshold. Further exploitation of the advantages of ultrasonographic dynamic measurements, such as shear-wave elastography and circulation flow measurements, will be needed for a more systematic approach to diagnose OSA patients. 

## 5. Conclusions

We can conclude that several upper airway or non-airway anatomical parameters measured using ultrasonography show good diagnostic accuracy, either alone or in combination with other demographic and anthropometric indices, in predicting OSA; these parameters also correlate well with AHI and other measures of disease severity and, thus, may have significant value for future screening of high-risk populations. Although the results are promising, more studies with larger samples and more standardized methodology are required before these measures are incorporated into clinical practice. Studies included in this review could serve as a basis for the development of new research pathways.

## Data Availability

No new data were created or analyzed in this study. Data sharing is not applicable to this article.
